# Is single-stage implant-based breast reconstruction (SSBR) with an acellular matrix safe? Strattice™ or Meso Biomatrix® in SSBR

**DOI:** 10.1007/s00238-018-1415-2

**Published:** 2018-04-24

**Authors:** Nadine S. Hillberg, Patrick I. Ferdinandus, Rieky E. G. Dikmans, Bjorn Winkens, Juliette Hommes, René R. W. J. van der Hulst

**Affiliations:** 1 Department of Plastic, Reconstructive and Hand Surgery, Maastricht University Medical Centre, Maastricht, The Netherlands; 2 Department of Plastic, Reconstructive and Hand Surgery, VU University Medical Centre, Amsterdam, the Netherlands; 3 Department of Methodology and Statistics, CAPHRI School for Public Health and Primary Care, Faculty of Health Medicine and Life Science, Maastricht, The Netherlands

**Keywords:** Acellular dermis, Breast implant, Breast reconstruction, Mesh matrix, Implant-based, Postoperative complications

## Abstract

**Background:**

Acellular matrices (AM) might enable a direct single-stage breast reconstruction procedure resulting in an improved efficacy of the reconstruction phase for patients. Safety concerns are an important issue due to a recent study which shows that single-stage breast reconstruction with Strattice™ resulted in more complications versus a two-stage reconstruction. Therefore, the goal of this study is to compare the short- and long-term complications of a single-stage breast reconstruction with the use of two types of AM (Strattice™ and Meso Biomatrix®) versus two-stage breast reconstruction without the use of an AM.

**Methods:**

Cohort study with single-stage breast reconstruction with Strattice™ (*n* = 28) or Meso BioMatrix® (*n* = 20) or two-stage breast reconstruction without an AM (*n* = 36) at the Maastricht Academic Hospital, the Netherlands. All complications, in particular major complications with the need for re-admission to the hospital, re-exploration, and implant explantation, were the primary outcome measures. A 1-year follow-up was achieved for all patients.

**Results:**

Baseline characteristics of all 52 patients were similar between groups. There was a significantly higher complication rate in the single-stage AM groups with loss of the implant in 40.0% of the breasts from the Meso BioMatrix® group and in 10.7% of the Strattice™ group compared to no implant loss in the control group.

**Conclusions:**

This cohort study clearly suggests that the use of a single-stage breast reconstruction is not safe with the use of these AMs. Well-designed prospective studies that guarantee the safety of those matrices should be published before these AMs are used in implant-based surgery.

Level of Evidence: Level III, risk / prognostic study.

## Introduction

Many women develop breast cancer and desire immediate breast reconstruction following mastectomy [1]. Implants are the most frequently performed breast reconstruction technique after skin sparing mastectomy [2, 3]. Due to insufficient muscular coverage for subpectoral placement of a breast implant after mastectomy, a tissue expander (TE) is inserted in the majority of patients. During outpatient consultations, the TE is expanded repeatedly to create sufficient room for the definitive breast implant.

For patient and cost-efficiency reasons, a single-stage implant-based breast reconstruction (SSBR) would be more desirable than a two-stage breast reconstruction (TSBR). However, single-stage surgery increases the risk of complications [1]. Since the introduction of acellular matrix (AM) meshes in 2005, a safe single-stage implant-based breast reconstruction technique using AM might be possible [4]. In SSBR, these meshes can provide sufficient coverage for a breast implant where the pectoralis and serratus muscle do not cover the implant if inserted directly after a skin-sparing mastectomy. These meshes could be the missing link to facilitate a SSBR.

**Fig. 1:**
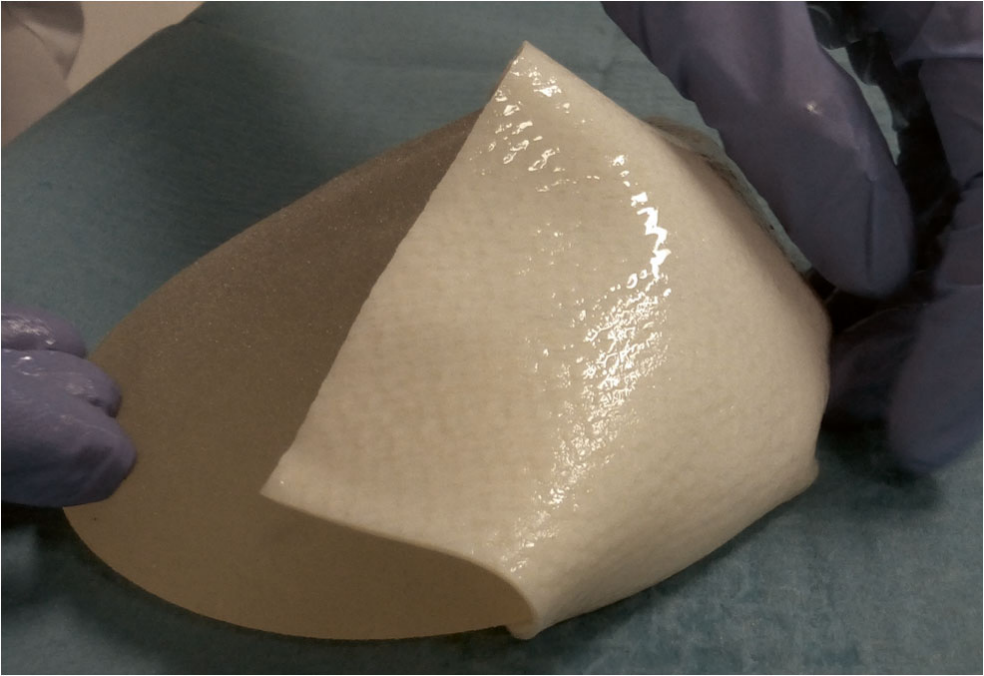
A photo illustrating placement of Strattice™ at the lower pole of a breast implant

**Fig. 2:**
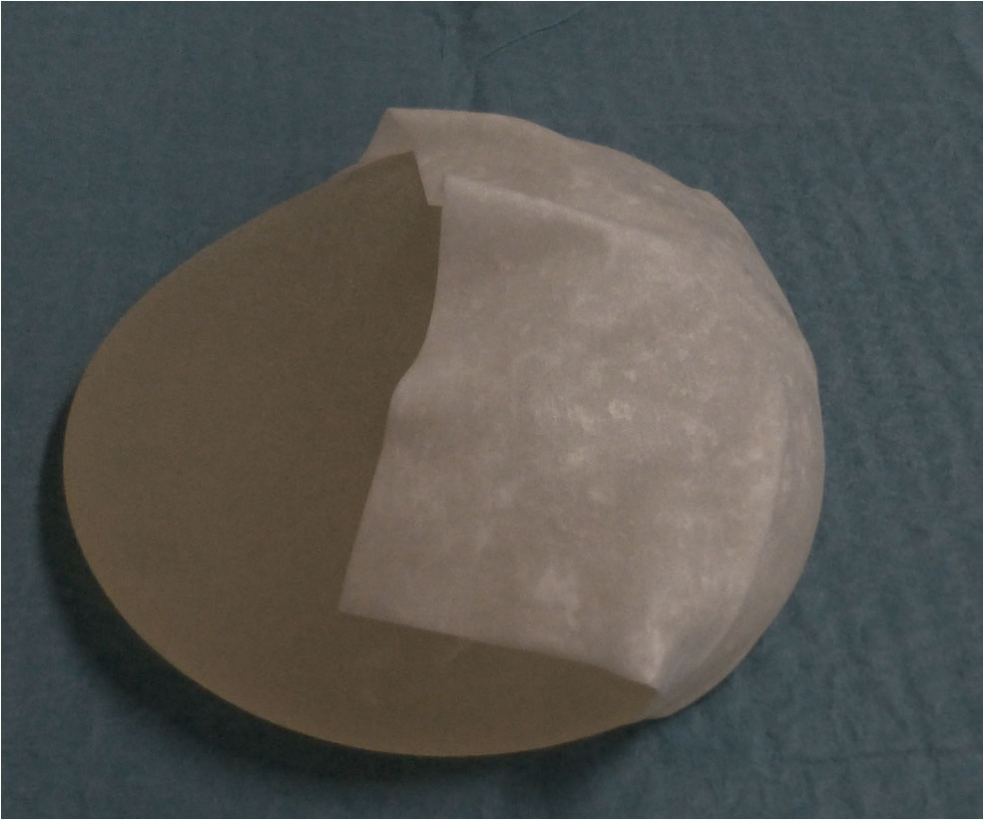
A photo illustrating placement of Meso BioMatrix® at the lower pole of a breast implant

Some studies report the use of an AM in the USA in more than 60% of all alloplastic reconstructions [5, 6]. Currently, several meshes are used including Strattice™, Alloderm®, and more recent the Meso BioMatrix® [7–9]. Up to now, studies have shown inconsistent results on the risks associated with the additional use of AM’s in both SSBR and TSBR [10–21]. Most studies summarize short-term safety using only one type of AM without a control group. Therefore, at the moment, there is no evidence which AM is the best. In 2013, a multicenter randomized clinical trial was started to compare the clinical outcomes and cost-effectiveness of a SSBR with Strattice™ with a TSBR without the use of AMs [22]. Our hospital included 40 patients for this study. After finishing the inclusion period for this study, Meso BioMatrix® entered the market and provided better matrix handling (Figs. 1 and 2), being more flexible and thin while maintaining strength. Therefore, patients who explicitly requested a single-stage procedure received a SSBR using Meso BioMatrix®.

The aim of this study was to assess the postoperative complications within 1 year associated with the use of Strattice™ and Meso BioMatrix® in implant-based breast reconstruction compared to a control group consisting of a TSBR without an AM.

## Patients and methods

### Study design

A single-center cohort study was done. Patients who participated in the BRIOS study at the Maastricht University Medical Centre were included [21]. These patients were randomly assigned into one of two groups. One group received immediate Strattice™ (LifeCell Corp., Branchburg, NJ, USA) assisted SSBR after mastectomy. The other group received an immediate, TSBR after mastectomy. Furthermore, a group of patients with immediate Meso BioMatrix® (DSM, Heerlen, The Netherlands) assisted implant-based breast reconstruction post-mastectomy was included. The latter group of patients did not take part in the BRIOS study and was not randomized.

The inclusion criteria for the BRIOS study met the inclusion criteria for the patients who received a breast reconstruction with the Meso BioMatrix®: women with BRCA gene mutation who underwent prophylactic treatment; women with a unifocal tumor smaller than 3–4 cm, intended to undergo a skin sparing mastectomy; willing and able to participate; aged 18 and over; and able to provide informed consent and able to complete questionnaires [21]. All patients where operated in the Maastricht University Medical Centre by one of the 12 plastic surgeons between May 2013 and March 2015.

Women with the following criteria were excluded in the BRIOS study as well in the Meso Biomatrix® group: a body mass index > 30 kg/m^2^; preferring a breast size larger than cup C; receiving a polyurethane implant; pregnant women; on-going severe psychiatric illness or mental retardation; evidence of alcohol and/or drug abuse; inability to complete questionnaires; a local or general infection which could jeopardize the surgical objective; an extensive local inflammatory reaction; proven or suspected hypersensitivity to materials; immunosuppressive pathologies; smokers; patients that would undergo postoperative radiotherapy.

### Outcome measures

Demographic information, co-morbid conditions, surgery details, and additional details, regarding breast cancer treatment, were obtained. The postoperative follow-up period for all patients was 1 year. A major complication was defined as any complication requiring hospitalization or surgical intervention with or without implant loss. Major complications requiring surgery with loss of the implant were defined as ‘explantation’. Complications that did not require hospitalization or surgery were defined as minor complications.

### Operative technique and postoperative management

The Strattice™ and control group received a single preoperative dose of cefazoline; in case of a penicillin allergy, erythromycin or clindamycin was given. The oncologic surgeon first performed a skin-sparing mastectomy before the plastic surgeon performed the SSBR or TSBR. A pocket was created under the major pectoral muscle to place the TE or implant. The cavity was cleaned with an antibiotic solution (cefaroxin 1000 mg/gentamicin 40 mg/betadine). During surgery, a ‘one touch’ technique of the implant was used and a ‘closed door’ policy was implemented to decrease the risk of infection. This means that the prosthesis or TE is touched once and only by the plastic surgeon and that the doors of the operating room remained closed in the period that the prosthesis was unpacked and handled by the plastic surgeon.

In the control group, the TE was placed in the pocket under the major pectoral muscle after which the major pectoral muscle was sutured together with the serratus/rectus fascia so the TE was fully covered by the muscle. After about 3 weeks, the TE will be expanded by multiple sessions of inflation with saline. The speed of inflation depended in particular on the size of the TE and the patient. Regular scheme is 50 cm^3^ perioperative fill and 50 cm^3^ per 2–3 weeks during the visit to the outpatient clinic. After the desired volume has been reached, the TE will be replaced for the final prosthesis in the second operation.

In the Strattice™ group, a sheet of Strattice™ was placed at the caudal side of the implant, sutured to the inferior margin of the musculus pectoralis major above and the inframammary crease below to complete the total pocket. Before use, the Strattice™ was washed in a saline solution as instructed by the manufacturer. In the Strattice™ group, two drains were placed: one anterior and one posterior to the matrix for at least 7 days. Postoperatively, the patients of the BRIOS study completed a 24-h regimen of cefazoline intravenously [21].

Because the Meso BioMatrix® group was not part of the BRIOS study, the operative technique was less standardized compared to the BRIOS study cases [21]. The Meso BioMatrix® group received, just as the BRIOS study, a single preoperative dose of cefazoline or same alternative in case of an allergy [21]. The ‘no touch’ and ‘closed door’ policy were similar, as was the operating technique to prepare a pocket and placing the Meso BioMatrix®. Before placement of the implant, the cavity and the wound-edges were cleaned with a betadine solution. The use of one or two drains differed between surgeons. The drains were removed with a production of less than 30 cm^3^/24 h per drain. Post-operative antibiotic prophylaxis consisted of another 5-day oral amoxicillin/clavulanic acid following a 24-h regimen of cefazoline intravenously.

All patients needed to wear a good fitting sports bra for at least 6 weeks post-operatively. The majority of patients were discharged from hospital with the drains in place. The drains were removed during a visit in the outpatient clinic or at home by nurses specialized in breast cancer care.

The patients included from the BRIOS study were randomized and treated following treatment protocol approved by the medical ethical committee [21]. The Meso Biomatrix® group consisted of a chart study. Therefore, this study was done in accordance with the Declaration of Helsinki and good clinical practice regulations.

### Statistical analysis

Statistical analyses were performed using IBM SPSS Statistics [23]. The differences in demographics between groups were assessed using a one-way ANOVA for continuous variables and Chi-square tests, or Fischer’s exact tests where appropriate for categorical variables. The proportion of complications within 1 year was compared between groups using logistic regression analysis. As a sensitivity analysis, the results were checked with generalized estimating equations (GEE) which accounts for a possible correlation between outcomes of breasts belonging to the same patient.

Since the expected number of patients with major complications was limited, the group effect was separately corrected for one of the potential confounders. As BMI, age, active smoking, radiotherapy, and adjuvant therapy influence the chance for complications of breast implant reconstructions, these were considered as potential confounders [1, 10, 16, 17, 24–27]. Major complications and explantations were combined, considering the expected low number of events. A *p* value ≤ 0.05 was considered to be statistically significant.

## Results

### Patient characteristics

In this study, 52 patients with 84 breasts were included. A 1-year follow-up was achieved for all patients. Twenty patients (38.5%) received a unilateral treatment and 32 patients (61.5%) received bilateral treatment. The reason for mastectomy was either prophylactic therapy in case of high hereditary risk for breast cancer (56%) or the presence of breast carcinoma (44%). Of all included patients, 21.4% of the breasts received a nipple-sparing mastectomy. A total of 15.5% of all breasts received an inframammary incision and 6% a partial peri areolar incision with a small extension lateral from the nipple. In one case in the TSBR, the tumor excision was incomplete with residual positive margins requiring re-excision.

The TSBR was performed in 36 breasts (43%), the SSBR using Strattice™ in 28 breasts (33%), and the SSBR with Meso BioMatrix® in 20 breasts (24%). In most cases, Eurosilicone implants (89.3%) were placed; Mentor (8.3%), Silimed (1.2%), and Allergan (1.2%) were used as well. All placed implants were textured. The mean volume of the placed implants was 370 cm^3^ for the Strattice™ group, 338 cm^3^ for the Meso BioMatrix® group, and 80 cm^3^ for the TE group. On average, six sessions (with variation from 4 till 11 sessions) of inflation were needed to reach the desired volume in the TE group.

Despite exclusion criteria for smoking and adjunctive breast radiation, 13.5% of the patients were active smokers during the perioperative period and 19.2% of the patients (10 breasts) received adjuvant radiotherapy; this was not significantly different between groups (Table 1). The start of the adjuvant radiotherapy differed between 28 and 192 days (mean 123 days) postoperatively. In six of these, a complication occurred. Except one case, all complications occurred before the start of the radiotherapy. In that one case, the breast received adjuvant radiotherapy 28 days postoperatively; then, 134 days later, an infection occurred with necrosis of the wound edge.

**Table 1:** Patient demographics of the different groups

	Control group	SSBR with Strattice™	SSBR with Meso BioMatrix®	*P* value *
Patients, *n*	21	19	12	
Age at operation, mean (SD), year	49.5 (10.9)	41.36 (11.7)	39.8 (13.9)	*0.040*
Smoking, *n* (% of patients)	3 (14.3%)	3 (15.8%)	1 (8.3%)	1.000^F^
BMI, mean (SD), kg/m^2^	21.8 (2.5)	22.7 (2.7)	21.1 (1.2)	0.199
DM, *n* (% of patients)	0	1 (5.3%)	0	0.596 ^F^
ASA 2, *n* (% of patients)	11 (50.0%)	7 (36.8%)	6 (50%)	0.641
Neo adjuvant therapy, *n* (% of patients)	2 (9.5%)	5 (26.3%)	2 (16.7%)	0.364 ^F^
Bilateral mastectomy, *n* (% of patients)	10 (47.6%)	11 (57.9%)	2 (16.7%)	0.073 ^F^
Lymph node dissection, *n* (% of patients)				
No lymph node resection	6 (28.6%)	5 (26.3%)	7 (58.3%)	
Sentinel lymph node resection	14 (66.7%)	10 (52.6%)	5 (41.7%)	
Axillary lymph node dissection	1 (4.8%)	4 (21.1%)	0	
Breasts, *n*	36	28	20	
Adjuvant therapy, *n* (% of patients)	10 (47.6%)	11 (57.9%)	2 (16.7%)	0.073 ^F^
Radiation, *n* (% of breasts)	4 (11.1%)	5 (17.9%)	1 (5.0%)	0.432 ^F^
Prophylactic mastectomy, *n* (% of breasts)	20 (55.6%)	14 (50.0%)	13 (65.0%)	0.586
Oncologic mastectomy, *n* (% of breasts)	16 (44.4%)	14 (50.0%)	7 (35.0%)	0.586
Nipple sparing, *n* (% of breasts)	4 (11.1%)	5 (17.9%)	9 (45.0%)	*0.016* ^F^
Mastectomy incision, *n* (% of breasts)				
Vertical mastectomy incision	32(88.9%)	23 (82.1%)	11 (55%)	
Inframammary incision	4 (11.1%)	2 (7.1%)	7 (35%)	
Partial peri areolar incision with a small extension lateral to the nipple	0	3 (10.7%)	2 (10%)	
Radical tumor excision	35 (97.2%)	28 (100%)	20 (100%)	1.000 ^F^
Eurosilicone implant placed *n* (% of breasts)	29 (80.6%)	27 (96.4%)	19 (95.0%)	0.115 ^F^
Definite implant volume cc, mean (SD)	379.7 (85.8)	370.0 (93.4)	337.5 (86.0)	0.229

*p value between groups were assessed by one-way ANOVA for numeric variables and Chi-square test or Fischer’s exact test for categorical variables. ^F^ = assessed by Fischer’s exact test

There was a statistically significant difference in age between the groups. The patients in the control group were older than in the Strattice™ or Meso BioMatrix® group. The patients in the Meso BioMatrix® group were on average younger, had a lower BMI, and fewer patients in this group received adjuvant chemotherapy and radiation therapy compared to the Strattice™ and control group. Thus on paper, the Meso BioMatrix® group was overall a more ideal patient group.

### Complications and re-interventions

**Fig. 3:**
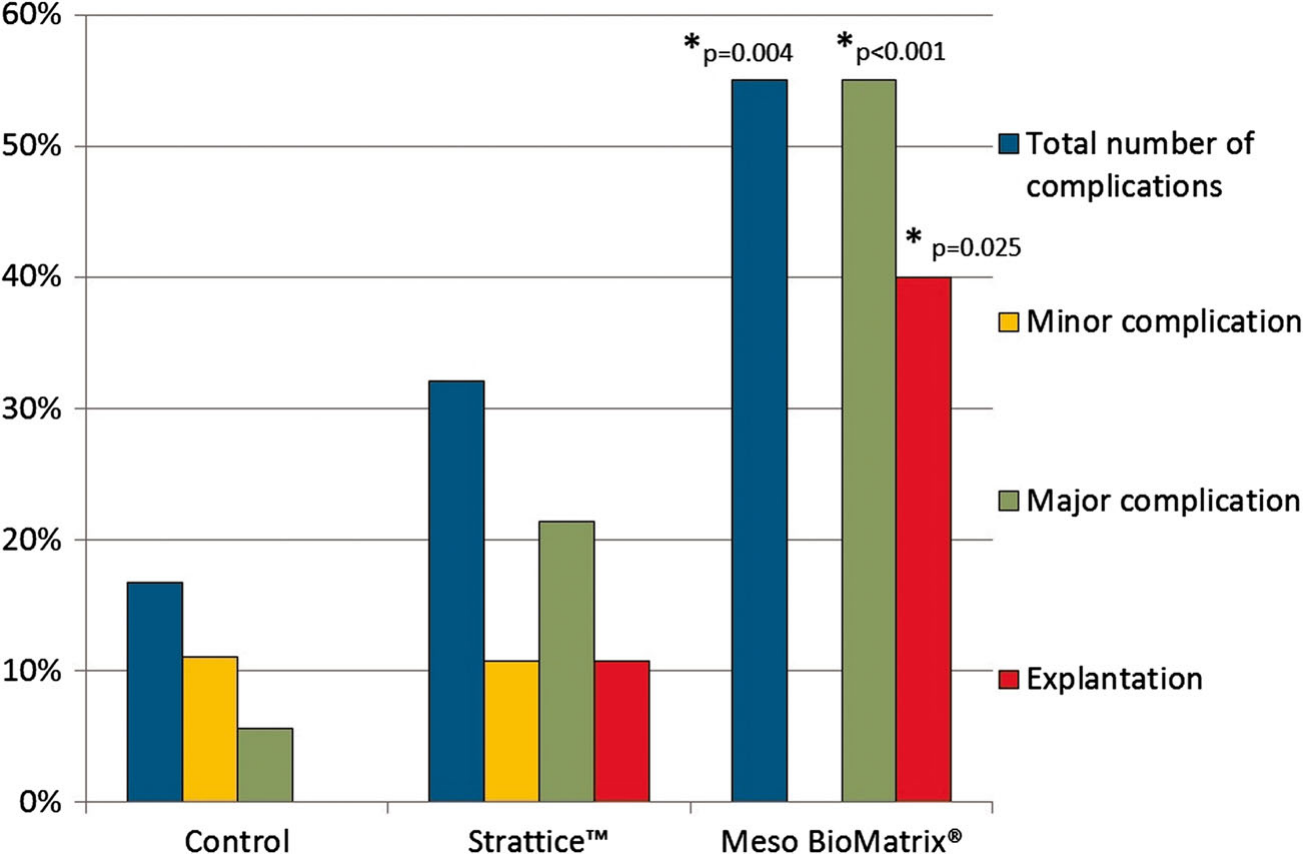
Illustrates complication rates among the three groups of patients included in this study. *Statistically significant compared to the control group

During the 1-year postoperative follow-up time, complications were encountered in 16.7% of the breasts of the control group, 32.1% of the Strattice™ group, and 55.0% of the Meso BioMatrix® group (Table 2, Fig. 3). Complications included wound infection, skin dehiscence, diathermal burn wound, hematoma, seroma, and skin ischemia. Major complications occurred in 55.0% of the breasts in the Meso BioMatrix® group, 21.4% of the breasts of the Strattice™ group, and 5.6% of the breasts of the control group. Explantation occurred in 40.0% of the breasts from the Meso BioMatrix® group and in 10.7% of the Strattice™ group compared to none in the control group. Patient demographics and reason of explantation are shown in Table 3.

**Table 2:** Complication rates between the groups

	Control (*n* = 36)	Strattice™ (*n* = 28)	Meso BioMatrix® (*n* = 20)
Total breasts with complications, *n* (%)	6 (16.7%)	9 (32.1%)	11 (55.0%)^*^
Infection	0	1 (1.3%)	5 (25%)
Hematoma	3 (8.3%)	0	1 (5%)
Necrosis of woundedge	1(2.8%)	5 (17.9%)	3 (15%)
Ischemic skin	0	0	2 (10%)
Edema	0	1 (3.5%)	0
Seroma	1 (2.8%)	2 (7.0%)	0
Postoperative bleeding	0	1 (3.5%)	0
Burnwound from ablation	1 (2.8%)	0	0
Dehiscence	1 (2.8%)	4 (14.3%)	1 (5%)
Minor *n* (%)	4 (11.1%)	3 (10.7%)	0
Major *n* (%)	2 (5.6%)	6 (21.4%)	11 (55.0%)^*^
Explantation *n* (%)	0(0.0%)	3(10.7%)^**^	8(40.0%)^**^

*Statistically significant (*p* < 0.05) compared to the control group

**Percentages of the groups Strattic™ or Meso Biomatrix®, respectively

**Table 3:** Per patient descriptive statistics if explantation occurred (*n* = 11)

Mesh	S	S	S	MB	MB	MB	MB	MB	MB	MB	MB
Age at operation, year	30	38	42	74	43	30	30	56	43	43	30
Smoking	No	No	Yes	No	No	No	No	Yes	?^a^	No	?^a^
BMI, kg/m^2^	22	23	22	23	20	21	21	22	23	23	19
DM	No	No	No	No	No	No	No	No	No	No	No
Neoadjuvant therapy	No	Yes	No	No	No	No	No	No	Yes	Yes	No
Bilateral mastectomy	Yes	No	Yes	No	No	Yes	Yes	Yes	Yes	Yes	Yes
Adjuvant therapy	Yes	Yes	No	No	No	No	No	No	No	No	No
Radiation	No	No	No	No	No	No	No	No	No	No	Yes
Prophylactic mastectomy	No	No	Yes	No	No	Yes	Yes	Yes	No	No	No
Oncologic mastectomy	Yes	Yes	No	Yes	Yes	No	No	No	Yes	Yes	Yes
Definitive implant volume, *cc*	370	410	410	290	435	320	320	515	410	410	320
Reason of explantation	EP	EP	EP	IN	EP	IE	IN	IN	EP	EP	EP
Time between operation and explantation, days	353	71	38	189	10	56	52	27	56	56	122

*S* Strattice™, *MB* Meso BioMatrix®, *EP* exposed prosthesis or mesh, *IS* ischemic skin, *IN* infection

^a^Missing

#### Meso BioMatrix® group versus control group

The Meso BioMatrix® group had a significantly higher complication rate than the control group (*p* = 0.004) (Table 3). After correcting for potential confounders, the results were similar and remained significant, irrespective of which potential confounder was corrected for. As for major complications, the same conclusion could be drawn with the effect being even larger (OR = 20.8; *p* < 0.001).

#### Strattice™ group versus control group

For total complications, major complications, and explantation, there were no significant differences in the Strattice™ group versus the control group (total complications: *p* = 0.153, major complications *p* = 0.075, explantation *p* = 0.079), irrespective of correcting for the potential confounders.

#### Meso BioMatrix® group versus Strattice™ group

The Meso BioMatrix® group showed significantly more major complications within 1 year compared to the Strattice™ group (*p* = 0.020). After correcting for the potential confounders, the results were similar and remained significant, irrespective of which potential confounder was corrected for (Table 4).

As for GEE analyses that accounts for the form of within-subject correlation if a patient had the breast reconstruction of both breasts, the results remained similar in all analyses and therefore yield the same conclusions.

## Discussion

Innovation is crucial in the field of plastic and reconstructive surgery. In this study, the use of acellular matrices (AMs) in implant-based breast reconstruction is reported using a single-stage breast reconstruction technique (SSBR). Postoperative complications between the two groups of patients with a SSBR with an AM, either Strattice™ made of porcine skin or Meso Biomatrix® made of porcine peritoneum, were compared to the two-stage implant-based breast reconstruction (TSBR). These groups of patients showed that the complication rate in the AM groups was much higher (32.1% in the Strattice™ group and 55.0% in the Meso BioMatrix® group) compared to the TSBR (16.7%) and significantly higher numbers of explantation (0 versus 10.7% in the Strattice™ group and 40.0% in the Meso Biomatrix® group). As AMs are widely used globally, it is of crucial importance to deliver high-quality evidence on the safety of these additional ‘implants’ inserted in patients. Registration in (inter)national independent opt-out implant registry could be of indispensable help in the prospective data sampling of all acellular (dermal) matrices to understand its implications for patient safety.

The amount of complications in the patients treated with a TSBR without a mesh was comparable to what is described in the literature [16, 28]. Nevertheless, the complication rates reported in the studies of Davila et al. and Collis et al. were much lower (5.4 and 7.4%) which might be due to a short follow-up (30 days) and not including minor complications with no need for further treatment [19, 29]. The higher amount of complications in the Strattice™ group compared to the control group has been replicated in the recently published randomized controlled trial described by Dikmans et al [22]. Our complication rate observed in the SSBR with the use Strattice™ is also comparable to what is described by Lardi et al [25]. However, the complication and explantation rate found in our study are significantly higher than the findings in Salzberg et al [8]. This could be the result of selection bias with a high inclusion rate of oncologic patients with adjuvant radiotherapy or (neo-) adjuvant chemotherapy.

The Meso BioMatrix® group experienced complications in 55.0% of the breasts and explantation in 40.0% of the breasts. Zero minor complications in the Meso Biomatrix® group suggests a detection bias of the cohort study, implying not precise enough reports of minor complications in the (outpatient) clinic. A prospective controlled study might thus reveal even higher complication rates in this Meso Biomatrix® group. To our knowledge, no studies have published the use of Meso BioMatrix® in SSBR yet. One trial is registered and is still including patients [30]. Therefore, the only comparison that can be made is with other types of AM [8, 11–13, 18, 24, 31]. In line with the previous studies, Meso BioMatrix® gives significantly more complications than described in other studies using AMs in a SSBR, where different kinds of AMs seem to have acceptable complication rates [8, 11–13, 18, 24, 31].

**Table 4:** Differences in total and major complication rate (within 1 year) between control group, Meso Biomatrix®, and Strattice™ group, after correction for no or one potential confounder

		Meso Biomatrix® versus control			Strattice™ versus control			Meso Biomatrix® versus Strattice™		
Total complications	Corrected for	OR	95%CI	*p* value	OR	95%CI	*p* value	OR	95%CI	*p* value
	–	6.1	1.8–21.2	0.004	2.4	0.7–7.7	0.153	2.6	0.8–8.4	0.117
	Age	10.0	2.1–47.9	0.004	2.5	0.7–9.4	0.163	3.2	0.9–11.5	0.079
	Smoking	6.7	1.7–26.3	0.007	2.5	0.7–8.1	0.143	2.8	0.8–10.7	0.123
	BMI	12.8	2.5–65.1	0.002	1.8	0.5–6.6	0.347	4.5	1.1–18.1	0.034
	Radiotherapy	8.2	2.1–31.9	0.003	2.2	0.6–7.6	0.206	3.4	0.9–12.0	0.061
	Adjuvant therapy	8.7	2.0–37.2	0.003	2.3	0.7–7.8	0.171	3.7	0.9–14.4	0.061
Major complications	–	20.8	3.9–111.1	<0.001	4.6	0.9–25.1	0.075	4.5	1.3–15.8	0.020
	Age	40.7	4.7–351.1	0.001	3.2	0.5–19.5	0.205	5.0	1.3–18.6	0.016
	Smoking	24.1	3.9–148.6	0.001	4.7	0.9–25.4	0.074	5.1	1.2–21.1	0.023
	BMI	37.7	4.4–319.8	0.001	3.8	0.7–21.5	0.128	7.2	1.6–32.4	0.010
	Radiotherapy	37.6	4.3–328.0	0.001	4.4	0.8–24.6	0.094	5.8	1.5–22.9	0.012
	Adjuvant therapy	42.8	4.4–416.5	0.001	4.8	0.8–28.1	0.080	8.9	1.7–47.0	0.010

Differences in complication rates in this study as well as in the literature could be explained by the mesh itself. Alloderm® is made out of human dermis; Strattice™ and Meso Biomatrix® are, respectively, obtained from porcine dermis and porcine mesothelial peritoneum. Instructions for use, the feel, and handling of these AMs are thus different, which assumes to also deem important for the result (function, esthetic, and complications) [32–34]. Interestingly, an SSBR with Alloderm® is suggested to have lower numbers of complications than generally found in the literature on SSBR and Strattice™ and especially compared to the complication rates found in this study. Hunsicker et al. recently published its 13 years of cumulative experience that showed a significantly lower complication rate compared to our study. In this study, several meshes were used of which 93% concerning Alloderm and only 6.9% Strattice and 0.1% FlexHD. These authors did not made a distinction in the complication rate per mesh, but found an overall complication rate of only 10% using an AM with SSBR [21]. Limitations in methodology (cohort/chart studies; single center, multiple surgeons, learning curve per surgeon); the general conclusion from all these studies is that we do not know how safe the use of an AM is, and how different complication rates between AMs are listed.

Seroma formation is a feared complication for surgeons, which might be higher when using AMs versus not using AMs. High rates of seroma formation have been described with the use of AM. In this study, we only observed three cases with seroma with one patient belonging to the control group (two-staged) and the other two in the Strattice group (9 and 25 days postoperative).

Questionable is to what extent the complications are caused by the single-stage procedure itself (versus for example patient characteristics) and whether the meshes play the pivotal role in this. Atiyeh et al. indicate that a SSBR without the use of a mesh could be done safely and that the final results largely depend on the status of the tissues after mastectomy [35, 36]. Colwell et al. also state that SSBR with or without using a mesh after nipple-sparing mastectomy could have a low complication rate if the patient is properly selected [37]. In an attempt to understand the role of AMs, we corrected for patient characteristics using as potential confounders including BMI, age at operation, adjuvant chemotherapy, radiotherapy, and active smoking behavior. The results were similar after correcting for these potential confounders separately. We have chosen these confounders based on possible risk factors for complications in implant-based breast reconstruction found in the literature [1, 10, 16, 24–27].

An important detail of this study was that complications that required explantation in both AM groups occurred rather late: in the Strattice™ group a median of 69 days (25–349 days) and in the Meso BioMatrix® group a median of 54 days (10–187 days). This emphasizes the need for a long follow-up when AMs are used. Barber et al. also reported the late occurrence of complications using a variety of AMs in breast reconstructive procedures with a median time until loss of implant of 73 days with a range of 9–895 days [10]. Currently, there is a lack of studies that examine the complications of AMs with a follow-up of several years. Moreover, studies seldom classify the time between implantation and complication occurrence although it is of significant importance for the outcome and of great interest for the doctor and the patient.

Limitations of this cohort study design are the risk of selection bias and detection bias in the Meso Biomatrix® group as explained earlier. Based on the patient demographics, however, the Meso BioMatrix® group contained a more favorable patient profile than the other two groups. The protocols of the two studies were slightly different from one another regarding the following: the postoperative prophylactic antibiotic regime, the amount of time that the drains were used, and the operative techniques. However, it is rather unlikely that these small variations explain the differences in complication rates between Strattice™ and Meso BioMatrix®. After all, most complications occurred in the Meso BioMatrix® group, despite the difference in drain- and antibiotic prophylaxis regime, which can be considered an advantage for the Meso BioMatrix® group. Considering that prolonged drain use can be associated with postoperative infection and although 24 h of prophylactic postoperative antibiotics seems equivalent to extended oral antibiotics for infections of TE-based breast reconstruction, it is not certain if this is also the case in SSBR with AMs [2, 38]. Nevertheless, despite of the limitations in the study, it does establish a strong suggestion.

All the surgeries were done in the same hospital performed by 12 plastic surgeons. In all three groups, the procedures were carried out by different plastic surgeons. The emergence of a complication did not seem to be associated with the plastic surgeon performing the operation. Insufficient soft tissue is an important aspect to predict complications. Unfortunately, there are no validated instruments to measure soft tissue quality. All surgeons would not place an implant if there were doubts on flap viability. Besides the complications, we did not compare possible improvement of appearance of the reconstructed breast or patient reported outcomes such as the quality of life between these groups. Even though the latter outcomes are rather important aspects of patient care, we are convinced that the first and foremost is the patient safety.

## Conclusion

This study suggests that the two-stage implant-based breast reconstruction without AMs is noticeably safer than the single-stage implant-based breast reconstruction with Strattice™ or Meso BioMatrix®. Before using an acellular (dermal) matrix in single-stage implant-based breast reconstructions, high-quality prospective studies on the safety of this procedure should be performed. An (inter)national implant registry could be of great value for post market surveillance of these innovations.
